# HIV status defines distinct immunological drivers of persistent portal hypertension after HCV cure in people with advanced cirrhosis

**DOI:** 10.3389/fimmu.2026.1683092

**Published:** 2026-02-02

**Authors:** Rubén Martín-Escolano, Amanda Fernández-Rodríguez, Laura Tarancon-Diez, Juan Berenguer, Helena Codina, Rafael Amigot-Sánchez, Juan González-García, Víctor Hontañón, Leire Pérez-Latorre, Luis Ibañez-Samaniego, Elba Llop-Herrera, Antonio Olveira, Laura Díaz, Isidoro Martínez, María Ángeles Jiménez-Sousa, Salvador Resino

**Affiliations:** 1 Unidad de Infección Viral e Inmunidad, Centro Nacional de Microbiología (CNM), Instituto de Salud Carlos III (ISCIII), Majadahonda, Madrid, Spain; 2 Centro de Investigación Biomédica en Red en Enfermedades Infecciosas (CIBERINFEC), Instituto de Salud Carlos III, Madrid, Spain; 3 Grupo de Infecciones en la Población Pediátrica, Instituto de Investigación Sanitaria del Gregorio Marañón, Madrid, Spain; 4 Unidad de Enfermedades Infecciosas/VIH; Hospital General Universitario “Gregorio Marañón”, Madrid, Spain; 5 Instituto de Investigación Sanitaria del Gregorio Marañón, Madrid, Spain; 6 Unidad de VIH, Servicio de Medicina Interna, Hospital Universitario “La Paz”, Madrid, Spain; 7 Servicio de Aparato Digestivo, Hospital General Universitario “Gregorio Marañón”, Madrid, Spain; 8 Centro de Investigación Biomédica en Red en Enfermedades Hepáticas y Digestivas (CIBEREHD), Instituto de Salud Carlos III, Madrid, Spain; 9 Departamento de Gastroenterología, Hospital Universitario Puerta de Hierro-Majadahonda, Majadahonda, Madrid, Spain; 10 Servicio de Aparato Digestivo, Hospital Universitario La Paz, Madrid, Spain; 11 Unidad de Citometría de Flujo y Sorter, Hospital General Universitario “Gregorio Marañón”, Madrid, Spain

**Keywords:** biomarkers, chronic hepatitis C, DAA therapy, HIV, HVPG regression, immunological profile

## Abstract

**Introduction:**

The immunological drivers of portal hypertension regression after hepatitis C virus (HCV) cure are poorly understood, particularly in the context of human immunodeficiency virus (HIV) coinfection We aimed to identify baseline immune signatures predicting the evolution of the hepatic venous pressure gradient (HVPG) in people with and without HIV (PWH/PWoH).

**Methods:**

We prospectively followed 41 individuals with advanced cirrhosis (18 PWoH, 23 PWH) who were cured of HCV with direct-acting antivirals (DAA). Baseline plasma and cellular immune markers were extensively profiled using multiplex assays and flow cytometry. We used mixed-effects modeling to test for associations between these baseline immune features and the change in HVPG over a 48-week follow-up period, with q-values controlling for false discoveries.

**Results:**

Two distinct immunological profiles of impaired HVPG regression emerged. In PWoH, impaired regression was linked to a broad proinflammatory profile [TNF-α (AMR = 1.13; q=0.012), IL17A (AMR = 1.28; q=0.012), and IL10 (AMR = 1.2; q=0.028)], a widespread total CD4+ T-cell activation [HLA-DR+ (AMR = 1.44; q<0.001) and CD38+HLA-DR+ (AMR = 1.3; q=0.007)], and robust activation across central memory (CM) and effector memory (EM) subsets. Conversely, in PWH, impaired HVPG regression was associated with sVCAM-1 (AMR = 1.58; q=0.096), and a more focused activation within EM (HLA-DR+, AMR = 1.08; q=0.030) and TemRA (CD38+HLA-DR+, AMR = 1.12; q=0.030) CD4+ T-cells.

**Discussion:**

HIV coinfection fundamentally reshapes the immunological landscape of post-cure portal hypertension recovery. The shift from systemic inflammation in PWoH to endothelial dysfunction and T-cell exhaustion in PWH reveals distinct pathological pathways. Understanding these signatures is a crucial step toward developing targeted therapies to promote complete hepatic recovery.

## 1 Introduction

Persistent portal hypertension is the primary driver of clinical outcomes in advanced cirrhosis, and it often remains unresolved despite successful hepatitis C virus (HCV) eradication with direct-acting antivirals (DAA) (1–3). The gold-standard measure for this residual risk is the hepatic venous pressure gradient (HVPG) (4). A substantial proportion of patients show incomplete hemodynamic regression following DAA therapy (5), which points to underlying host factors perpetuating liver injury. Understanding these factors is crucial for post-cure risk stratification.

The persistent portal hypertension is attributed to a residual “immunological scar” that outlives the virus. At the systemic level, this scar manifests as a proinflammatory environment, driven by fibrogenic cytokines like tumor necrosis factor-alpha (TNF-α) and Interleukin (IL)-17A (6). At the cellular level, the T-cell compartment remains deeply altered, with features of exhaustion, senescence (e.g., expansion of CD57+ T-cells), and a loss of functional memory populations, all of which are established drivers of liver fibrosis (7–10).

This landscape is particularly amplified in the context of human immunodeficiency virus (HIV) coinfection. HIV infection exhibits a more profound and persistent state of chronic immune activation, characterized by heightened co-expression of markers like human leukocyte antigen-DR (HLA-DR) and CD38, even with effective antiretroviral therapy (ART) (6, 11, 12). This creates a distinct inflammatory milieu that likely alters the fundamental drivers of liver repair (9, 13, 14), raising the critical question of whether the immunological determinants of HVPG regression in coinfected individuals mirror those in monoinfection.

This study aimed to identify the baseline immunological determinants of HVPG regression after DAA therapy in people with advanced HCV-related cirrhosis. By analyzing a comprehensive panel of both plasma and T-cell biomarkers, we sought to delineate the distinct pathological profiles associated with incomplete liver recovery in individuals with and without HIV coinfection.

## 2 Materials and methods

### 2.1 Study design and participants

This prospective, multicenter study evaluated 41 individuals with HCV-related advanced cirrhosis, comprising 18 people without HIV (PWoH) and 23 with HIV (PWH). Participants were enrolled from four tertiary hospitals in Madrid, Spain, throughout 2015 as part of the ESCORIAL study (see **Appendix** for details). The study protocol was conducted in accordance with the Declaration of Helsinki and approved by the Research Ethics Committee of the Instituto de Salud Carlos III (CEI PI 41_2014-v2). All participants provided written informed consent prior to inclusion.

Inclusion criteria required participants to have active HCV infection (detectable HCV-RNA) at pre-treatment time-point (baseline) and to subsequently achieve a sustained virological response (SVR) at 12 weeks post-treatment with DAAs. Additionally, all individuals had a formal diagnosis of advanced cirrhosis and data, including baseline plasma samples and HVPG measurements at both the pre-treatment and 48-week post-DAA therapy time points.

For inclusion, advanced cirrhosis was defined by at least one of the following: LSM ≥25 kPa, Child-Pugh score ≥7, HVPG ≥10 mmHg, or a history of a major decompensation event (e.g., ascites, variceal bleeding, or hepatic encephalopathy).

### 2.2 Clinical assessments and study definitions

Comprehensive clinical data were prospectively collected and managed using a secure electronic data capture system. Hemodynamic studies to assess the HVPG were conducted on fasted participants under light midazolam sedation, following established protocols (15). Liver stiffness measurement (LSM) was performed by experienced operators using transient elastography (FibroScan^®^, Echosens, Paris, France), with results reported in kilopascals (kPa) (16). The Child-Pugh score was determined using standard clinical and laboratory parameters (17).

### 2.3 Endpoint and explanatory variables

The primary endpoint was the HVPG regression, assessed at baseline and the 48-week post-treatment follow-up. The main explanatory variables were the baseline concentrations of plasma inflammatory biomarkers and the frequencies of T-cell subsets.

### 2.4 Sample collection

Peripheral blood was collected from study participants into BD Vacutainer™ tubes with Hemogard™ closures containing K3-ethylenediaminetetraacetic acid (EDTA) (Becton Dickinson, Franklin Lakes, NJ, USA). Subsequently, plasma was recovered, carefully collected, aliquoted and stored at -80°C at the Spanish HGM BioBank, following density gradient centrifugation using Ficoll-Paque™ PLUS (Cytiva, Uppsala, Sweden).

### 2.5 Multiplex immunoassays

Baseline plasma concentrations of key cytokines and chemokines were determined using a custom ProcartaPlex™ multiplex immunoassay (Bender MedSystems GmbH, Vienna, Austria) on a Luminex 200™ platform (Bio-Rad Laboratories, Hercules, CA, USA). The assay was performed following the manufacturer’s protocol. Briefly, plasma samples were clarified by centrifugation (10,000 x g for 10 min at 4°C) to remove particulates and diluted as required. Magnetic beads conjugated with capture antibodies were incubated with the samples, standards, and blanks in a 96-well black flat-bottom plate overnight at 4°C with shaking (500 rpm). After washing, biotinylated detection antibodies were added and incubated for 30 min at room temperature. Subsequently, a streptavidin-phycoerythrin (SAPE) reporter was added for 30 min at room temperature. After final washing steps, the beads were resuspended in reading buffer, and the fluorescent signal was acquired on the Luminex 200™ instrument using Bio-Plex Manager™ software. The panel was designed to quantify Th1/Th2/Th17 cytokines (interferon (IFN)-γ, IL-12p70, IL-2, IL-10, IL-4, IL-17A), inflammatory mediators (IL-1β, IL-18, IL-6, TNF-α), and endothelial dysfunction markers (soluble vascular cell adhesion molecule-1 (sVCAM-1) and soluble intercellular adhesion molecule-1 (sICAM-1)). For the analysis, we utilized raw median fluorescence intensity (MFI) values as a direct measure of analyte abundance, a validated approach that enhances statistical power by including low-signal samples without imposing a limit of detection (18, 19).

### 2.6 Flow-cytometry

Multiparametric flow cytometry was utilized to perform detailed CD4 and CD8 T-cell immunophenotyping on 100 µL of fresh anticoagulated whole blood at baseline, as previously described (16). Samples were stained with a custom panel of antibodies targeting surface markers CD3, CD4, CD8, CD45RA, CD28, CD38, HLA-DR, and CD57 (see **Supplementary Table 1** for details). Following a 20-minute incubation at room temperature in the dark, red blood cells were lysed and leukocytes were fixed using the IMMUNOPREP Reagent System on a Coulter MULTI-Q-PREP Lysing Workstation (Beckman Coulter, Miami, FL, USA). Data were acquired on a Gallios™ flow cytometer (Beckman Coulter, Brea, CA, USA), collecting at least 200,000 lymphocyte events per sample. Data analysis, conducted with Kaluza™ software, involved first gating on CD3+ lymphocytes, which were then separated into CD4+ and CD8+ T-cell lineages. These populations were further stratified into four differentiation subsets based on CD45RA and CD28 expression: Naïve (CD45RA+CD28+), central memory (CM; CD45RA-CD28+), effector memory (EM; CD45RA-CD28-), and terminally differentiated (TemRA; CD45RA+CD28-). Finally, the functional state of these subsets was assessed by quantifying the percentage of cells expressing markers of immune activation (CD38, HLA-DR) and senescence (CD57).

(CD57).

### 2.7 Statistical analysis

Baseline cohort characteristics were compared using the Mann-Whitney U test (continuous variables) and the χ² or Fisher’s exact test (categorical variables).

To evaluate the association between baseline biomarkers and the longitudinal change in HVPG, we used Generalized Linear Mixed-Effects Models (GLMMs), an approach well-suited for repeated-measures data. Each model was specified with a gamma distribution and a log link, appropriate for skewed, positive continuous data like HVPG. A random intercept for each participant accounted for the correlation between repeated measurements. The model’s fixed effects included the natural log-transformed (ln-transformed) baseline biomarker, the time point (visit: baseline vs. follow-up), HIV status, and all their corresponding interaction terms. The primary term of interest was the three-way interaction (biomarker × visit × HIV status), which formally tests whether the association between a biomarker and HVPG change over time differs significantly between the two HIV strata. From this unified model, we then calculated the stratum-specific effects for PWH and PWoH using linear combinations of the model coefficients. These effects are presented as the Arithmetic Mean Ratio (AMR), which represents the multiplicative change in the post- to pre-treatment HVPG ratio for each one-unit increase in the ln-transformed biomarker. An AMR >1 indicates an association with impaired regression (higher residual HVPG), while an AMR <1 indicates an association with improved regression (lower residual HVPG).

To control the false discovery rate (FDR) across the analyses, p-values for the stratum-specific AMRs were adjusted using the Benjamini-Hochberg procedure. This adjustment was applied independently within three pre-defined biomarker families: plasma cytokines, CD4+ T-cell subsets, and CD8+ T-cell subsets. Associations were considered statistically significant at an FDR-adjusted p-value (q-value) of <0.10, a standard threshold for identifying robust signals in exploratory biomarker research.

All statistical analyses were performed using Stata version 17.0 (StataCorp, TX, USA) and SPSS version 25.0 (SPSS Inc., Chicago, IL, USA).

## 3 Results

### 3.1 Characteristics of the study population

As detailed in **Table 1**, the PWoH and PWH cohorts exhibited distinct baseline profiles. PWH were younger (p<0.001), more frequently male (p=0.011), and had higher rates of past intravenous drug use (p<0.001). They had lower rates of prior interferon-based therapy (p=0.004) and better-preserved liver function, as reflected by a lower Child-Pugh score (p=0.016). Despite these differences, the groups did not differ significantly in HVPG, ensuring their comparability for the primary analysis.

**Table 1:** Summary of baseline characteristics of people with advanced HCV-related cirrhosis, stratified by HIV status.

Variable	HVPG progression evaluation		
	PWoH	PWH	*p*
No.	18	23	
Gender (male)	8 (44.4%)	19 (82.6%)	**0.011**
Age (years)	62.8 (52.9 - 66.3)	52.1 (48.7 - 53.2)	**<0.001**
Smoker			0.101
Never	7 (38.9%)	3 (13.0%)	
Previously (≥ 6 months)	4 (22.2%)	4 (17.4%)	
Nowadays	7 (38.9%)	16 (69.6%)	
Alcohol intake			0.347
Never	13 (72.2%)	13 (56.5%)	
Previously (≥ 6 months)	5 (27.8%)	8 (34.8%)	
Nowadays	0 (0.0%)	2 (8.7%)	
Intravenous drug use			<0.001
Never	16 (88.9%)	7 (30.4%)	
Previously (≥ 6 months)	2 (11.1%)	16 (69.6%)	
BMI (kg/m^2^) (n=36)	27.0 (25.6 - 31.2)	24.5 (22.9 - 27.3)	0.064
BMI ≥ 25 kg/m^2^	11 (78.6%)	9 (40.9%)	0.067
Treatments			
Previous HCV therapy (IFNα+rib)	15 (83.3)	9 (39.1)	**0.004**
Statins	17 (94.4)	18 (78.3)	0.146
Antiretroviral therapy			
NRTI+NNRTI-based	–	2 (8.7%)	–
NRTI+II-based	–	12 (52.2%)	–
NRTI+PI-based	–	4 (17.4%)	–
PI+II+others-based	–	3 (13%)	–
Others	–	2 (8.7%)	–
HIV markers			
Prior AIDS	–	8 (34.8%)	–
Nadir CD4+ T-cells (cells/mm^3^)	–	99 (54 - 207)	–
Nadir CD4+ <200 cells/mm^3^	–	17 (73.9%)	–
CD4+ T-cells (cells/mm^3^)	–	490 (234 - 721)	–
CD4+ <500 cells/mm^3^	–	13 (56.5%)	–
HCV markers			
HCV genotype (n=40)			0.224
1	15 (83.3%)	13 (56.5%)	
3	2 (11.1%)	3 (13.0%)	
4	1 (5.6%)	6 (26.1%)	
Log_10_ HCV-RNA (IU/mL)	6 (5.6 - 6.3)	6.2 (5.5 - 6.7)	0.462
HCV-RNA ≥850,000 IU/mL	8 (44.4%)	10 (43.5%)	0.951
Liver disease			
LSM (kPa)	32.1 (21.3 - 61.5)	35.3 (22.3 - 41.6)	0.486
<25 kPa	5 (27.8%)	6 (26.1%)	0.359
25–40 kPa	5 (27.8%)	11 (47.8%)	
≥40 kPa	8 (44.4%)	6 (26.1%)	
Child-Pugh Score (n=38)	5 (6 - 7)	5 (5 - 5)	**0.016**
Child-Pugh ≥7	5 (33.3%)	3 (13%)	0.134
Decompensated cirrhosis	11 (61.1%)	8 (34.8%)	0.093
HVPG (mmHg)	17 (14.5 - 18.5)	15 (11.0 - 17.5)	0.188
<16 mmHg	8 (44.4%)	13 (56.5%)	0.459
16–20 mmHg	6 (33.3%)	8 (34.8%)	
>=20 mmHg	4 (22.2%)	2 (8.7%)	

Statistics: Values expressed as absolute number (percentage) and median (interquartile range). P-values, raw p-values; q-values, p-values corrected for multiple testing using the false discovery rate (Benjamini and Hochberg) procedure. The statistically significant differences are shown in bold. AIDS, acquired immune deficiency syndrome; BMI, body mass index; HCV, hepatitis C virus; HIV, human immunodeficiency virus; IFNα, interferon-alpha; NRTI, nucleoside analogue HIV reverse transcriptase inhibitor; NNRTI, non-nucleoside analogue HIV reverse transcriptase inhibitor; PI, protease inhibitor; II, integrase inhibitor; HCV-RNA, HCV plasma viral load; HVPG, hepatic venous pressure gradient; LSM, liver stiffness measurement; PWH, people with HIV; PWoH, people without HIV; rib, ribavirin.

### 3.2 Plasma biomarkers define distinct systemic profiles

The association between baseline plasma biomarkers and HVPG regression was fundamentally different depending on HIV status (**Table 2**). In PWoH, impaired HVPG regression was robustly linked to a systemic proinflammatory signature. After correcting for multiple comparisons, higher baseline levels of TNF-α (AMR = 1.13; q=0.012), IL-17A (AMR = 1.28; q=0.012), and the immunoregulatory cytokine IL-10 (AMR = 1.20; q=0.028) all remained significantly associated with poorer outcomes. Conversely, in PWH, the only significant associated factor was sVCAM-1 (AMR = 1.58; q=0.096), indicating that endothelial dysfunction, rather than systemic inflammation, was the key driver of poor outcomes in this cohort.

**Table 2:** Association between baseline plasma biomarkers and HVPG regression after 48 weeks of completing HCV treatment in people with HCV-related advanced cirrhosis.

	PWoH			PWH		
Plasma biomarkers	AMR (95%CI)	p-value	q-value	AMR (95%CI)	p-value	q-value
Th1/Th2/Th17 cytokines						
IFNγ	1.08 (1.00; 1.17)	**0.050**	0.150	1.03 (0.72; 1.47)	0.865	0.865
IL12p70	1.12 (0.93; 1.35)	0.246	0.428	1.17 (0.87; 1.57)	0.293	0.600
IL2	1.12 (0.91; 1.38)	0.285	0.428	1.12 (0.61; 2.04)	0.723	0.838
IL10	1.20 (1.05; 1.38)	**0.007**	**0.028**	0.89 (0.77; 1.03)	0.121	0.600
IL4	1.10 (0.91; 1.34)	0.325	0.433	1.15 (0.86; 1.52)	0.345	0.600
IL17A	1.28 (1.10; 1.50)	**0.002**	**0.012**	1.27 (0.77; 2.12)	0.350	0.600
Inflammatory cytokines						
IL1β	1.03 (0.99; 1.06)	0.136	0.326	1.07 (0.68; 1.67)	0.768	0.838
IL18	1.00 (0.94; 1.07)	0.911	0.911	0.92 (0.78; 1.08)	0.307	0.600
IL6	1.06 (0.95; 1.18)	0.274	0.428	0.96 (0.88; 1.03)	0.256	0.600
TNFα	1.13 (1.05; 1.21)	**0.001**	**0.012**	1.08 (0.72; 1.62)	0.710	0.838
Endothelial dysfunction						
sVCAM1	1.07 (0.55; 2.09)	0.840	0.911	1.58 (1.12; 2.22)	**0.008**	**0.096**
sICAM1	1.02 (0.90; 1.16)	0.741	0.889	1.06 (0.92; 1.23)	0.412	0.618

Statistics: Data are presented as Arithmetic Mean Ratio (AMR) with 95% Confidence Intervals (95%CI) and p-values, derived from a generalized linear mixed-effects model (gamma family, log link). The AMR represents the multiplicative change in the ratio of post- to pre-treatment HVPG for each one-unit increase in the log-transformed baseline marker. An AMR > 1 indicates an association with impaired regression (higher residual HVPG), while an AMR < 1 indicates an association with improved regression (lower residual HVPG). Q-values are p-values adjusted for multiple comparisons using the False Discovery Rate (FDR) procedure, as developed by Benjamini and Hochberg. Values in bold highlight statistically significant associations after correction for multiple comparisons (q-value<0.10) or significant unadjusted p-values (p-value<0.05). AMR, Arithmetic Mean Ratio; CI, Confidence Interval; HVPG, hepatic venous pressure gradient; IFNγ, interferon-gamma; IL, interleukin; PWH, people with HIV; PWoH, people without HIV; sICAM-1, soluble intercellular adhesion molecule-1; sVCAM-1, soluble vascular cell adhesion Molecule-1; TNFα, tumor necrosis factor-alpha.

### 3.3 Divergent CD4+ T-cell signatures of impaired regression

Distinct CD4+ T-cell profiles were associated with HVPG regression, with patterns diverging significantly based on HIV status (**Table 3**). In PWoH, impaired HVPG regression was linked to a broad signature of immune dysregulation (AMR>1, q <0.1). This included activation of total CD4+ T-cells (HLA-DR+, q<0.001; CD38+HLA-DR+, q=0.007) and a larger population of EM CD4+ T-cells (q=0.020). The association was particularly strong within the CM CD4+ compartment, where the levels of activation (HLA-DR+, q =0.015; CD38+HLA-DR+, q=0.007) and senescence (CD57+, q=0.074) markers all associated with a poorer outcome. In a critical counterpoint, a larger baseline CM CD4+ T-cell population was the sole factor associated with improved regression (AMR = 0.75; q=0.074), suggesting a protective role for this memory subset. However, the profile in PWH was more restricted. Impaired HVPG regression was specifically linked to activated EM CD4+ T-cells (HLA-DR+, q=0.030) and TemRA CD4+ T-cells (CD38+HLA-DR+, q=0.030).

**Table 3:** Association between baseline CD4+ T-cell subsets (percentage) and HVPG regression (mmHg) 48 weeks after completing HCV treatment.

		PWoH			PWH		
T-cell Subset	Marker	AMR (95% CI)	p-value	q-value	AMR (95% CI)	p-value	q-value
**Total**	Base Population	–	–		–	–	
*(CD4+)*	CD38+	1.12 (0.98; 1.28)	0.094	0.187	1.12 (0.76; 1.65)	0.576	0.883
	HLA-DR+	1.41 (1.20; 1.67)	**<0.001**	**<0.001**	1.19 (1.02; 1.39)	**0.031**	0.155
	CD38+ HLA-DR+	1.30 (1.11; 1.52)	**0.001**	**0.007**	1.08 (0.92; 1.28)	0.344	0.860
	CD57+	1.03 (0.93; 1.15)	0.510	0.678	0.92 (0.74; 1.14)	0.435	0.883
**Naïve**	Naïve	1.23 (0.97; 1.56)	0.095	0.187	0.94 (0.70; 1.25)	0.662	0.883
**Central Memory (CM)**	CM	0.75 (0.58; 0.97)	**0.026**	**0.074**	1.15 (0.76; 1.73)	0.507	0.883
*(CD4+CD45RA-CD28+)*	CD38+	1.14 (0.97; 1.32)	0.103	0.187	0.97 (0.78; 1.20)	0.771	0.903
	HLA-DR+	1.25 (1.08; 1.45)	**0.003**	**0.015**	1.07 (0.89; 1.28)	0.455	0.883
	CD38+ HLA-DR+	1.22 (1.09; 1.38)	**0.001**	**0.007**	0.93 (0.70; 1.25)	0.647	0.883
	CD57+	1.16 (1.02; 1.33)	**0.026**	**0.074**	0.82 (0.61; 1.10)	0.189	0.540
**Effector Memory (EM)**	EM	1.08 (1.02; 1.14)	**0.005**	**0.020**	0.98 (0.88; 1.09)	0.719	0.899
*(CD4+CD45RA-CD28-)*	CD38+	1.03 (0.94; 1.12)	0.578	0.680	0.96 (0.83; 1.10)	0.538	0.883
	HLA-DR+	1.01 (0.92; 1.12)	0.837	0.863	1.08 (1.03; 1.14)	**0.003**	**0.030**
	CD38+ HLA-DR+	0.95 (0.86; 1.04)	0.230	0.354	1.06 (0.99; 1.12)	0.088	0.330
	CD57+	1.02 (0.84; 1.23)	0.863	0.863	1.00 (0.87; 1.15)	0.986	0.986
**TemRA**	TemRA	1.04 (0.91; 1.19)	0.542	0.678	1.01 (0.92; 1.11)	0.813	0.903
*(CD4+CD45RA+CD28-)*	CD38+	1.11 (0.99; 1.25)	0.081	0.187	0.98 (0.76; 1.28)	0.905	0.953
	HLA-DR+	1.09 (0.97; 1.23)	0.166	0.277	1.10 (0.98; 1.24)	0.099	0.330
	CD38+ HLA-DR+	1.03 (0.91; 1.16)	0.667	0.741	1.12 (1.04; 1.20)	**0.002**	**0.030**
	CD57+	0.98 (0.92; 1.04)	0.463	0.661	1.08 (1.01; 1.15)	**0.023**	0.153

Statistics: Data are presented as Arithmetic Mean Ratio (AMR) with 95% Confidence Intervals (95%CI) derived from a generalized linear mixed-effects model (gamma family, log link). The AMR represents the multiplicative change in the ratio of post- to pre-treatment HVPG for each one-unit increase in the log-transformed baseline marker. An AMR > 1 indicates an association with impaired regression (higher residual HVPG), while an AMR < 1 indicates an association with improved regression (lower residual HVPG). Only markers with a statistically significant association (p < 0.05) in at least one stratum are shown. Q-values are p-values adjusted for multiple comparisons using the False Discovery Rate (FDR) procedure, as developed by Benjamini and Hochberg. Values in bold highlight statistically significant associations after correction for multiple comparisons (q-value<0.10) or significant unadjusted p-values (p-value<0.05). AMR, Arithmetic Mean Ratio; CD, Cluster of Differentiation; CI, Confidence Interval; HLA-DR, Human Leukocyte Antigen – DR; HVPG, hepatic venous pressure gradient; PWoH, people without HIV; PWH, people with HIV; TemRa, Terminally Differentiated Effector Memory.

### 3.4 Limited role of the CD8+ T-cell compartment

While CD4+ T-cell findings revealed notable associations, baseline CD8+ T-cell profiles, conversely, were not significantly linked to HVPG regression after correcting for multiple comparisons (**Supplementary Table 2**). This indicates that the CD8+ T-cell compartment plays a limited, if any, role in modulating HVPG recovery in this setting.

## 4 Discussion

In this prospective study of patients with advanced cirrhosis cured of HCV, we demonstrate that the immunological drivers of impaired regression (higher residual HVPG) are fundamentally different depending on HIV status. Our findings delineate two distinct pathological hallmarks. In PWoH, persistent portal hypertension was characterized by a dual signature of systemic inflammation, led by TNF-α, and broad CD4+ T-cell activation. In stark contrast, the profile in PWH shifted away from this classic inflammatory axis. Here, incomplete regression was linked to endothelial dysfunction, marked by sVCAM-1, and a more focused activation restricted to the memory CD4+ T-cell compartment.

Although DAA therapy achieves high HCV cure rates, persistent immune dysregulation is thought to drive the ongoing risk of liver disease progression in patients with advanced HCV- related cirrhosis (8, 9, 20–22). Our findings on baseline plasma biomarkers provide compelling, population-specific evidence for this hypothesis, highlighting distinct pathways of injury.

In PWoH, a pre-existing proinflammatory state was a key determinant of impaired HVPG regression. Specifically, elevated baseline levels of TNF-α, IL-17A, and IL-10 were robustly associated with a worse outcome. The roles of TNF-α and IL-17A in promoting liver injury are well-established; TNF-α directly activates hepatic stellate cells (HSCs) to drive fibrogenesis (23), while IL-17A orchestrates tissue inflammation and damage (24). The concurrent association with IL-10, a cytokine with known anti-inflammatory properties, is particularly revealing. Rather than indicating a protective response, its elevation in this proinflammatory context likely signifies a state of profound immune dysregulation, where its homeostatic functions are overwhelmed or ineffective (25). This triad of biomarkers thus paints a picture of a self-perpetuating inflammatory loop that hinders hepatic recovery.

In PWH, this classic inflammatory signature lost its prognostic value. Here, the primary systemic driver of impaired HVPG regression shifted entirely to endothelial dysfunction, with sVCAM-1 emerging as the sole significant plasma biomarker. This finding is critical, as sVCAM-1 is a well-established marker of endothelial activation and vascular injury, previously linked to liver disease severity in the context of HIV (9, 26, 27). We hypothesize that this mechanistic shift is driven by the pervasive, high-grade inflammation characteristic of PWH. This intense inflammatory “background noise”, fueled by factors like microbial translocation, likely obscures the more subtle, organ-specific cytokine signals relevant in PWoH. Consequently, the constant stress on the vascular endothelium emerges as a more dominant and measurable driver of pathology. The unique association with sVCAM-1 thus reflects a fundamental divergence where pathways of chronic vascular injury supplant cytokine-driven inflammation, a critical distinction for managing post-SVR risk in this population.

Our detailed T-cell immunophenotyping provides a cellular basis for the divergent pathways of persistent liver injury, revealing a distinct “immunological scar” whose features are dictated by HIV status.

In PWoH, impaired HVPG regression was associated with a broad CD4+ T-cell dysregulation, characterized by widespread activation (HLA-DR+, and CD38+HLA-DR+ co-expression) across total, CM, and EM CD4+ T-cell subsets. This suggests that a history of prolonged antigenic stimulation from HCV leaves behind a dysfunctional, proinflammatory CD4+ T-cell environment that acts as a key barrier to hepatic recovery (28, 29). Critically, however, this detrimental profile was counterbalanced by a significant protective factor: a robust baseline population of CM CD4+ T-cells was independently associated with improved regression. This novel finding highlights a crucial dichotomy: while T-cell activation perpetuates injury, the capacity for functional, long-term immunity, embodied by the CM pool, is essential for effective liver regeneration post-cure.

In PWH, the immunological landscape was markedly different. While CD4+ T-cell activation remained a key driver of poor outcomes, it was more restricted to differentiated memory subsets (EM and TemRA cells). More importantly, the protective signature of a healthy CM CD4+T-cell pool was absent in the coinfected group, suggesting a more profound and uncompensated immune exhaustion. We hypothesize this is due to the unique pressures of HIV infection, which establishes a state of non-specific, systemic immune activation fueled by factors like microbial translocation (11). This environment promotes widespread “bystander activation,” driving T-cells into premature exhaustion and depleting the restorative CM subset. Since these drivers are intrinsic to HIV and not resolved by HCV cure, this dysfunctional CD4+ T-cell landscape provides a clear mechanistic explanation for the impaired liver recovery observed in this cohort (30).

In stark contrast to the robust associations observed within the CD4+ lineage, we found no significant links between any baseline CD8+ T-cell phenotype and post-cure HVPG regression. This does not necessarily imply that CD8+ T-cells are irrelevant to liver pathology, but rather suggests that their baseline peripheral state is not a primary determinant of long-term hemodynamic recovery. It is plausible that the key immunological battleground for hepatic regeneration resides within the helper and regulatory functions of the CD4+ T-cell compartment, which orchestrates the overall inflammatory tone and tissue repair processes.

Taken together, our cellular findings point towards the CD4+ T-cell axis as the central mechanism governing incomplete liver recovery. The nature of this involvement—broad but counterbalanced in PWoH versus focused and uncompensated in PWH—and the absence of a restorative immune reservoir in the coinfected group, define the distinct routes to persistent portal hypertension post-SVR.

This study has several notable strengths. Its prospective, multicenter design, specifically enrolling patients with advanced cirrhosis, enhances the clinical applicability of our findings to this high-risk population. From a methodological standpoint, the use of a repeated-measures design analyzed via GLMMs provided a powerful framework for precisely modeling longitudinal changes in HVPG. Furthermore, the concurrent evaluation of a comprehensive panel of both plasma and fresh cellular biomarkers allowed for an analysis of the immunological environment, revealing distinct, population-specific pathways.

However, certain limitations must be acknowledged. The primary limitation is the modest sample size, which, particularly after stratification by HIV status, limited our statistical power to detect associations of smaller magnitude. This may explain the lack of significant findings within the CD8+ T-cell compartment and constrain our ability to perform more complex multivariate analyses. Second, as an observational study, we can report associations but cannot infer causality, and the potential for residual confounding from unmeasured variables remains. Finally, our assessment was restricted to a single 48-week follow-up after completing HCV therapy. While this provides a valuable snapshot of the long-term outcome, it does not capture the complete dynamic trajectory of how HVPG regression unfolds over time; future studies with intermediate time points would be beneficial.

In conclusion, HIV coinfection fundamentally reshapes the immunological landscape of post-cure portal hypertension recovery in advanced HCV-related cirrhosis. The shift from a systemic inflammatory profile in PWoH to one dominated by endothelial dysfunction and a more exhausted T-cell phenotype in PWH reveals distinct, non-overlapping pathological hallmarks. Understanding these distinct signatures is the first step toward developing targeted therapies to promote complete hepatic recovery post-cure.

## Data Availability

The original contributions presented in the study are included in the article/**Supplementary Material**. Further inquiries can be directed to the corresponding author.
