# Supplementary material for: HIV status defines distinct immunological drivers of persistent portal hypertension after HCV cure in people with advanced cirrhosis
Source: Front Immunol. 2026 Feb 2;17:1683092. doi: 10.3389/fimmu.2026.1683092 (PMC12907311; doi:10.3389/fimmu.2026.1683092)
Supplement: Supplementary file 1 [file Table1.docx]

Supplementary Material

# Supplementary Table 1. List of monoclonal antibodies used for flow cytometry.

| **Marker** | **Fluorochrome** | **Clone** | **Manufacturer** |
| --- | --- | --- | --- |
| CD3 | Pacific Orange (PO) | UCHT1 | Invitrogen |
| CD4 | APC-Cyanine 7 (APC-Cy7) | OKT4 | BioLegend |
| CD8 | Pacific Blue (PB) | SK1 | BioLegend |
| CD45RA | Phycoerythrin-Texas Red (ECD) | 2H4LDH11LDB9 | Beckman Coulter |
| CD28 | Phycoerythrin (PE) | CD28.2 | Beckman Coulter |
| CD38 | APC-Cyanine 5 (APC-Cy5) | HIT2 | BioLegend |
| HLA-DR | Allophycocyanin (APC) | L243 | BioLegend |
| CD57 | Fluorescein (FITC) | HCD57 | BioLegend |

# Supplementary Table 2. Association between baseline CD8+ T-cell subsets (percentage) and HVPG regression (mmHg) after HCV treatment.

|  |  | **PWoH** | | | **PWH** | | |
| --- | --- | --- | --- | --- | --- | --- | --- |
| **T-cell Subset** | **Marker** | **AMR (95% CI)** | **p-value** | **q-value** | **AMR (95% CI)** | **p-value** | **q-value** |
| **Total** | Base Population | - | - |  | - | - |  |
| *(CD8+)* | CD38+ | 0.96 (0.85; 1.10) | 0.588 | 0.843 | 0.81 (0.65; 1.02) | 0.070 | 0.324 |
|  | HLA-DR+ | 1.11 (0.99; 1.25) | 0.084 | 0.545 | 1.05 (0.88; 1.26) | 0.556 | 0.654 |
|  | CD38+ HLA-DR+ | 1.09 (0.95; 1.25) | 0.224 | 0.560 | 0.82 (0.59; 1.14) | 0.240 | 0.400 |
|  | CD57+ | 0.90 (0.72; 1.13) | 0.377 | 0.685 | 0.78 (0.6; 1.02) | 0.065 | 0.324 |
| **Naïve** | Base Population | 1.11 (0.95; 1.30) | 0.206 | 0.560 | 1.07 (0.77; 1.47) | 0.701 | 0.738 |
| **Central Memory (CM)** | Base Population | 0.95 (0.75; 1.20) | 0.667 | 0.889 | 1.21 (0.93; 1.56) | 0.152 | 0.324 |
| *(CD8+CD45RA-CD28+)* | CD38+ | 1.03 (0.92; 1.16) | 0.572 | 0.843 | 0.84 (0.70; 1.00) | 0.054 | 0.324 |
|  | HLA-DR+ | 1.11 (1.00; 1.23) | **0.050** | 0.545 | 1.04 (0.87; 1.24) | 0.698 | 0.738 |
|  | CD38+ HLA-DR+ | 1.11 (0.99; 1.24) | 0.079 | 0.545 | 0.90 (0.78; 1.05) | 0.178 | 0.324 |
|  | CD57+ | 0.98 (0.75; 1.28) | 0.856 | 0.984 | 0.83 (0.63; 1.07) | 0.153 | 0.324 |
| **Effector Memory (EM)** | Base Population | 1.00 (0.91; 1.11) | 0.986 | 0.986 | 0.90 (0.78; 1.05) | 0.173 | 0.324 |
| *(CD8+CD45RA-CD28-)* | CD38+ | 1.00 (0.89; 1.11) | 0.952 | 0.986 | 0.89 (0.74; 1.05) | 0.169 | 0.324 |
|  | HLA-DR+ | 1.09 (0.98; 1.20) | 0.109 | 0.545 | 1.06 (0.96; 1.18) | 0.261 | 0.402 |
|  | CD38+ HLA-DR+ | 1.08 (0.97; 1.21) | 0.157 | 0.560 | 0.92 (0.72; 1.17) | 0.493 | 0.616 |
|  | CD57+ | 0.96 (0.77; 1.20) | 0.722 | 0.903 | 0.74 (0.51; 1.06) | 0.095 | 0.324 |
| **TemRA** | Base Population | 0.88 (0.69; 1.11) | 0.283 | 0.629 | 0.88 (0.69; 1.12) | 0.293 | 0.419 |
| *(CD8+CD45RA+CD28-)* | CD38+ | 0.97 (0.87; 1.08) | 0.590 | 0.843 | 0.91 (0.76; 1.1) | 0.338 | 0.451 |
|  | HLA-DR+ | 1.08 (0.96; 1.21) | 0.187 | 0.560 | 1.13 (0.95; 1.34) | 0.156 | 0.324 |
|  | CD38+ HLA-DR+ | 1.06 (0.94; 1.19) | 0.341 | 0.682 | 0.99 (0.80; 1.23) | 0.942 | 0.942 |
|  | CD57+ | 0.98 (0.72; 1.32) | 0.886 | 0.984 | 0.78 (0.63; 0.97) | **0.025** | 0.324 |

**Statistics**: Data are presented as Arithmetic Mean Ratio (AMR) with 95% Confidence Intervals (95%CI) derived from a generalized linear mixed-effects model (gamma family, log link). The AMR represents the multiplicative change in the ratio of post- to pre-treatment HVPG for each one-unit increase in the log-transformed baseline marker. An AMR > 1 indicates an association with impaired regression (higher residual HVPG), while an AMR < 1 indicates an association with improved regression (lower residual HVPG). Only markers with a statistically significant association (p < 0.05) in at least one stratum are shown. Q-values are p-values adjusted for multiple comparisons using the False Discovery Rate (FDR) procedure, as developed by Benjamini and Hochberg. Values in bold highlight statistically significant associations after correction for multiple comparisons (q-value<0.10) or significant unadjusted p-values (p-value<0.05). **Abbreviations**: HVPG, hepatic venous pressure gradient; PWoH, people without HIV; PWH, people with HIV; AMR, Arithmetic Mean Ratio (AMR); 95%CI, 95% Confidence Intervals.
